# Content/Potency Assessment of Botulinum Neurotoxin Type-A by Validated Liquid Chromatography Methods and Bioassays

**DOI:** 10.3390/toxins11010035

**Published:** 2019-01-12

**Authors:** Bruna Xavier, Rafaela Ferreira Perobelli, Maurício Elesbão Walter, Francielle Santos da Silva, Sérgio Luiz Dalmora

**Affiliations:** 1 Postgraduate Programme in Pharmaceutical Sciences; Federal University of Santa Maria, Santa Maria 97105-900, Brazil; bxavier28@gmail.com (B.X.); rafaelaperobelli@gmail.com (R.F.P.); mauricio.walter13@gmail.com (M.E.W.); fransantos.biomed@gmail.com (F.S.d.S.); 2 Industrial Pharmacy Department, Federal University of Santa Maria, Santa Maria 97105-900, Brazil

**Keywords:** botulinum neurotoxin type A, size-exclusion chromatography, reversed-phase chromatography, T−47D cell culture, LD_50_ mouse bioassay

## Abstract

Botulinum neurotoxin type-A (BoNTA) is one of the seven different serotypes (A to G) produced by Clostridium botulinum. A stability-indicating size-exclusion chromatography (SEC) method was developed and validated, and the specificity was confirmed by forced degradation study, interference of the excipients, and peaks purity. The method was applied to assess the content and high-molecular-weight (HMW) forms of BoNTA in biopharmaceutical products, and the results were compared with those of the LD_50_ mouse bioassay, the T−47D cell culture assay, and the reversed-phase chromatography (RPC) method, giving mean values of 0.71% higher, 0.36% lower, and 0.87% higher, respectively. Aggregated forms showed significant effects on cytotoxicity, as well as a decrease in the bioactivity (*p* < 0.05). The employment of the proposed method in conjunction with the optimized analytical technologies for the analysis of the intact and altered forms of the biotechnology-derived medicines, in the correlation studies, enabled the demonstration of the capability of each one of the methods and allowed for great improvements, thereby assuring their safe and effective use.

## 1. Introduction

Type-A toxin is one of the seven different serotypes (A to G) currently known to be produced by *Clostridium botulinum*, and it is the first to receive attention clinically [1]. Botulinum neurotoxin type-A (BoNTA) acts by blocking the release of acetylcholine at the neuromuscular junction, which prevents muscle contraction. BoNTA is most widely used for aesthetic and therapeutic applications, such as cervical dystonia, blepharospasm, spastic conditions, pain, hyperhidrosis, and migraines [2,3,4].

BoNTA is a polypeptide that contains 1296 amino acids and has a molecular mass of 150 kDa; it is cleaved by proteases to generate the active di-chain form of the toxin, which is linked by a single disulfide bond at positions (C_430_–C_454_) [5,6]. The 50-kDa light chain acts as a metalloprotease that prevents the release of acetylcholine from the peripheral nerve terminals. The 100-kDa heavy chain contains two functional domains, the C-terminal part is the receptor-binding domain, and the N-terminal part is responsible for the translocation of the light chain across the membrane of the intraneuronal vesicles into the cytosol [7].

Bioassays are quantitative procedures based on functional responses in living systems and have been used for the potency assessment of biological products, and the products derived from bacterial toxins are also evaluated using in vivo assays. The LD_50_ mouse bioassay is currently the gold standard method to estimate the potency of BoNTA, as it evaluates all four of the functions necessary for toxicity (binding to the cell, uptake, translocation from the vesicle, and enzymatic activity) [8,9,10]. This assay requires a large number of animals, and is time-consuming and expensive. Thus, substantial effort has been spent to find alternatives, following the principles of the refinement, reduction, and replacement (3 Rs) [11,12]. Promising in vitro endopeptidase assays and cell-based assays using different cell lines and end-point detection have been explored in parallel with alternative in vivo models. Then, the challenges posed by the validation process performed following the international recommendations are essential for establishing alternatives to replace the mouse bioassay for potency determination [13,14].

The production of BoNTA requires the assessment of the biological potency, and the measurement of the enzymatic activity of the light chain can be used as an alternative approach for the in vitro assay, but is performed in association with a procedure that evaluates the heavy chain properties. A SNAP-25 chip-based assay using surface plasmon resonance with the detection of specific proteolytic products was developed showing the potential to monitor the activity of pharmaceutical preparations of BoNTA [15,16]. In vitro assays have been developed based on the binding of BoNTA to antibodies, or on the proteolytic activity in endopeptidase assays [17]. The effect of BoNTA that induces caspase-3- and caspase-7-dependent apoptotic processes, with inhibitory effects on the proliferation of the cell line T-47D, was used for evaluating the responses with 3-(4,5-dimethylthiazol-2-yl)-2,5- diphenyltetrazolium bromide (MTT) [18]. A new assay based on the inhibitory activity of BoNT on the stimulus-dependent release of a luciferase from a differentiated human neuroblastoma-based reporter cell line, was suggested in order to measure the biological activity of the pharmaceutical preparations of BoNTA and bacterial neurotoxins [19]. Biological, immunological, and physicochemical methods used for *Clostridium botulinum* and its toxins were reviewed, mentioning their limitations and their usefulness for the detection and diagnostic [20]. However, currently, there is no international biological reference standard for BoNTA; therefore, each manufacturer uses a unique product reference for the evaluation of bioactivity as specific units, which are not interchangeable, and the products are not standardized across manufacturers [21].

Physicochemical methods are essential in order to ensure certain quality aspects of clinical-grade proteins, but as they cannot yet predict their bioactivity, a combination of analytical technologies is recommended [22]. Liquid chromatography (LC) methods have proven to be useful for monitoring the content, purity, identity, and chemical stability of biotechnology-derived medicines [23,24,25]. Size-exclusion chromatography (SEC) has become the most widely applied technique for the analysis of native-proteins and for their aggregates, which can be generated during fermentation, purification, and processing, and have the potential to elicit an immunoresponse and may affect the biological activity [26,27]. Reversed-phase chromatography (RPC) methods were applied to assess the related proteins as well as the main peak in biopharmaceutical formulations, combined with mass spectrometry, for the detection of soluble *N*-ethylmaleimide-sensitive factor attachment protein receptor (SNARE) cleavage products generated from formulated BoNTA [28,29].

This research aimed to develop and validate a specific SEC method in order to evaluate the content of BoNTA in biotechnology-derived medicines; assess the bioactivity of their high-molecular-weight (HMW) forms; and evaluate the correlation of the results with the LD_50_ bioassay, the T-47D assay, and the RPC method. In this context, the current work is an extension of the previously published literature [28], contributing to assuring the quality and clinical efficacy of this biotherapeutic.

## 2. Results and Discussion

### 2.1. In Vitro Cell Culture Assay

The experimental conditions of the in vitro bioassay were investigated by changing the cell concentrations within 2 × 10^5^ to 4.5 × 10^5^ cells mL^−1^, and the 3 × 10^5^ cells mL^−1^ showed an improved discrimination related to the cells’ control. Then, the incubation time was tested between 4 to 6 h, selecting 5 h because of the higher reproducibility of the absorbances. Moreover, the time of exposure of the cells to BoNTA was tested at 18, 24, and 48 h, so as to determine the cell response, and was optimized as 24 h. Then, the optimized conditions were used to select the doses of BoNTA in the linear region of the dose-response curve, between 3 and 81 U mL^−1^.

### 2.2. Development of the SEC Method

**Figure 1:**
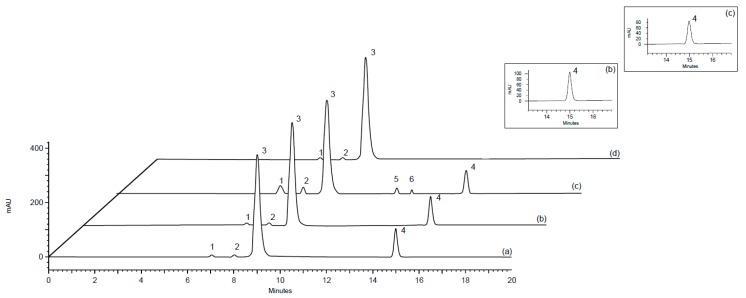
Size-exclusion chromatography (SEC) chromatograms showing peak 1 and 2 = Aggregated forms from human serum albumin (HSA); peak 3 = HSA; peak 4 = botulinum neurotoxin type-A (BoNTA); peaks 5 and 6 = high-molecular-weight (HMW) proteins. (**a**) Biological reference substance of botulinum neurotoxin type-A (BRS-BoNTA), (**b**) pharmaceutical product Botox^®^, (**c**) after stress at 60 °C for 8 h and shaking for 1 h, (**d**) placebo.

The chromatographic method was developed by testing mobile phases containing potassium and sodium phosphate, sodium acetate, sodium chloride, and phosphoric acid, at a concentration range of 0.05 M to 0.4 M, and at pH from 4 to 7.5. The 50 mM potassium phosphate solution at pH 7.0 enabled better sensitivity and a shorter retention time of BoNTA. Additionally, the mobile phase composition was tested by adding small amounts from 5% to 10% (*v*/*v*) of organic modifiers—isopropanol and methanol—however, the effects observed in the separation were non-significant. Then, the columns (300 and 600 mm lengths) with different porosity were evaluated, and the TSKgel^®^ G3000 SW_XL_ provided a higher efficiency and a shorter retention time. Furthermore, a photodiode array (PDA) detector was used to select the 220 nm wavelength, which corresponds to the maximum absorbance of BoNTA [30]. Representative chromatograms, as obtained for the three products, using the selected parameters, are illustrated in Figure 1a,b, with the peaks relative to the BoNTA detected at 15 min.

### 2.3. Validation of the SEC Method

The stress testing condition showed the specificity of the method that exhibited a significant decrease of the monomer peak area (22%) eluting at 15 min, and two other peaks that were observed at 12.0 and 12.7 min (Figure 1c), which were attributed to the HMW species of the protein. Moreover, the analysis of a formulation containing only human serum albumin (HSA) and sodium chloride, demonstrated three peaks at 7.0, 8.0, and 9.2 min, which were identified as aggregate forms and HSA (Figure 1d). The peak purity was checked by monitoring the chromatographic eluates with the PDA detector, demonstrating that they were considered homogeneous, with a peak purity index >0.9999, indicating a complete separation without interfering peaks; thereby, this performance suggests that the SEC method is suitable for pharmaceutical analysis.

**Figure 2:**
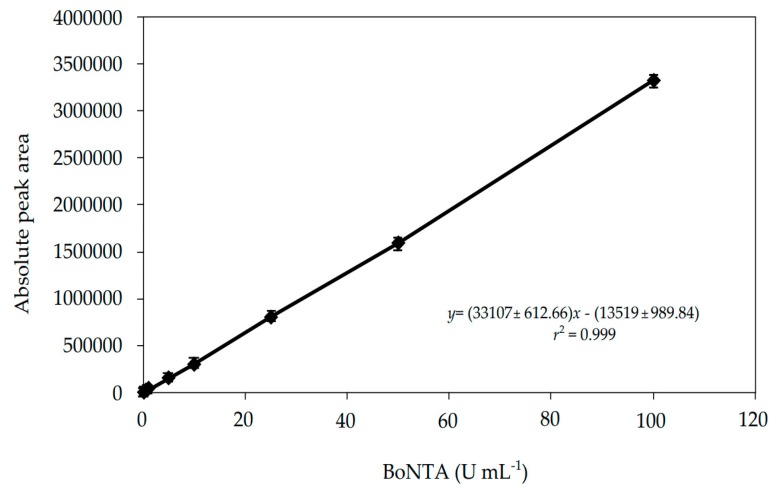
Analytical curve constructed for the SEC method.

The linear range of the method was verified by analyzing the biological reference substance of botulinum neurotoxin type-A (BRS−BoNTA) concentrations (*n* = 8) in triplicate, and constructing three independent analytical curves against the absolute peak area responses. The regression analysis was calculated using the least-squares method, using the STATGRAPHICS Centurion XVII Version 17.2.05 software (Warrenton, VA, USA), demonstrating a linearity in the range of 0.29–100 U mL^−1^ (Figure 2). The y-intercept was not-significantly different from zero (*p* > 0.05), and the slope differed significantly from zero (*p* < 0.05).

The limit of detection (DL) and the limit of quantitation (QL) were calculated from the slope and the standard deviations of the intercept of the analytical curves, giving values of 0.08 and 0.29 U mL^−1^, respectively. The QL was also determined experimentally as 0.25 U mL^−1^, with an accuracy within ±5% [31].

The intraday precision was determined as the repeatability, evaluating the concentrations of BoNTA (40, 50, and 60 U mL^−1^), and the relative standard deviations (RSD%) were calculated as 0.35%, 0.20%, and 0.25%, respectively. The interdays precision was tested, and two samples were analyzed on three consecutive days, giving an RSD of 0.35% and 0.62%. The between-analysts precision was also assessed, yielding RSD results of 0.60% and 0.11%.

The accuracy of the method was determined by spiking the placebo with known concentrations of BoNTA, to prepare solutions corresponding to 80%, 100%, and 120%, of the working solution. The analysis showed an absolute mean of 100.41%, and a bias <0.93% (Table 1).

The robustness was investigated by varying the chromatographic conditions, as given in Table 2, demonstrating acceptable results (RSD ≤2%), and did not show any significant differences (*p* > 0.05). The stability of the BoNTA solutions was also assessed, showing stability for 24 h (98.18%, RSD% 0.76) in the auto-sampler and for 48 h at 2–8 °C (99.80%, RSD% 0.19), with non-significant differences relative to the freshly prepared samples.

The performance of the analytical system was assessed using the suitability test, and the RSD% calculated from five replicates of a BRS−BoNTA solution (50 U mL^−1^), for the retention time, peak area, and peak symmetry were 0.05%, 0.47%, and 0.32%, respectively. The peak capacity (k) and the number of theoretical plates were calculated as 5.49 and 21,120.16, respectively. All of the parameters measured were according to the limits recommended [32].

### 2.4. Application of the LC Methods and Bioassays

The validated SEC method was applied to quantitate the BoNTA in the pharmaceutical preparations, and the results were compared to those obtained from the in vivo and in vitro bioassays and from the RPC method previously studied [28], giving mean differences of an estimated content of 0.71% higher, 0.36% lower, and 0.87% higher, respectively, with non-significant differences (*p* > 0.05) (Table 3). Moreover, a significant correlation was demonstrated for the SEC method with the in vivo mouse lethality assay (Pearson’s correlation coefficient *r* = 0.9984). The results of the analytical methods studied also demonstrated that, because of the varying capabilities, a combination of technologies is necessary to characterize, monitor the instability, and evaluate the content/potency of the biopharmaceutical products from different manufacturers.

In addition, a sample was subjected to a stressed condition at 60 °C for 8 h and shaking for 1 h, and was analyzed using the SEC method, and the results showed HMW forms (Table 4) and significant degradation (*p* < 0.05). Then, the sample was subjected to the in vitro bioassay, which demonstrated a significant decrease in the bioactivity to 29.10% (Table 4, 4^e^). Thus, the bioactivity was tested by the in vivo bioassay, and no toxic effects or mortality of the mice were detected. In addition, the bioactivity of the related proteins detected using the RPC method, although previously assessed after 4 h of exposure to UV light [28], was tested again so as to evaluate the effects after 8 h, which demonstrated little decrease from 98.70% (197.40 U mL^−1^) to 95.20% (190.40 U mL^−1^) (Table 4, 4^f^). The biological activity was generated by the interaction of the protein with its target receptor, and, as observed, the effects of the aggregated forms evaluated by the in vitro assay showed differences related to those of the LD_50_ mouse bioassay data. The therapeutic or toxic actions of BoNTA were thought to be mediated only by enzymatic SNAP-25 cleavage. However, additional toxin effects detected by the in vitro and in vivo findings suggest the existence of other mechanisms of action related to neuroexocytosis, cell cycle and apoptosis, neuritogenesis, and gene expression, which should be investigated, as previously described [33]. Then, the in vitro cell culture bioassay was performed based on the antiproliferative and cytotoxic effects using the cell line T-47D, but, in order to understand the complex mechanisms involved, additional studies are recommended. The potential of each one of the methods was demonstrated, and their employment in conjunction represents improvements in terms of developing alternative analytical methods to characterize the biotechnology-derived medicine.

### 2.5. Cytotoxicity Evaluation

The intact molecules and degraded samples were subjected to the cytotoxicity test, giving means of inhibitory concentration 50 (IC_50_) = 3.35 ± 0.86 U mL^−1^ and 4.23 ± 0.22 U mL^−1^, and IC_50_ = 1.53 ± 0.11 U mL^−1^ and 1.32 ± 0.19 U mL^−1^, respectively, for mouse connective tissue NCTC clone 929 and Chinese hamster ovary (CHO) cell lines. The differences were significant, as calculated by the Student’s t-test (*p* < 0.05). The test showed lower toxic effects on the cells compared with the intact biomolecule. A reduction of the toxic effects was also observed using the in vivo mouse lethality assay. Because of the inherent complexity and for safety reasons, such evaluations may represent a contribution to the concerns about possible undesirable human effects resulting from the instability of the pharmaceuticals during production and storage [34].

## 3. Conclusions

The validated SEC method and the correlation of the results demonstrated by the methods investigated for different pharmaceutical products, contribute to establishing alternatives in the context of 3Rs. Moreover, the current research has made advances in characterization, and allows for advances that can be used during the biotechnology processes and purification phases, to monitor the instability, and to assure batch-to-batch quality and consistency of this biotherapeutic.

## 4. Materials and Methods

### 4.1. Reagents and Chemicals

Products of Botox^®^ (Allergan, Inc., Irvine, CA, USA), Botulift^®^ Bergamo (Medytox Inc., Cheongwon-gu, Korea), and Botulim^®^ Blau Farmacêutica (Hugel, Inc., Chuncheon, Korea) containing 100 and 200 U vial^−1^ within their shelf-life period were purchased from commercial sources. The batches and expiry dates were as follows: C3566C3F February 2017; FAB1601 January 2019; FAA1605 January 2019; HUA17120 September 2019; FAA17021 March 2020; C4321C3 September 2019; FAA17010 February 2020. The biological reference substance of botulinum neurotoxin type-A (BRS−BoNTA) was obtained from Medytox Inc. (Seocho-Gu, Seoul City, Korea), as a toxin complex lyophilized powder, and was stored at −20 °C. Dulbecco’s modified Eagle’s medium (DMEM), RPMI 1640 medium, foetal bovine serum (FBS), dimethyl sulfoxide, neutral red, and the alamarBlue^TM^ cell viability reagent were purchased from Sigma-Aldrich (St. Louis, MO, USA). Human serum albumin (HSA); sodium acetate; sodium chloride; phosphoric acid; sodium phosphate; potassium phosphate monobasic; and the HPLC-grade solvents for chromatography, acetonitrile, methanol, and isopropanol were purchased from Merck (Darmstadt, Germany). The water was purified using the Milli-Q A10 system from Millipore (Bedford, MA, USA).

### 4.2. Equipments

The viable cells were assessed quantitatively using absorbance measurements [35] on a Varioskan^®^ Flash microplate reader from Thermo Scientific (Vantaa, Finland).

Chromatographic separations were performed using an analytical system, which consisted of a system controller, a quaternary pump, an autosampler, a column oven, and PDA detector from Shimadzu (Kyoto, Japan). The LC Solution Version 1.22 SP1 software was used for the instrument control, data acquisition, and analysis. The chromatographic mobile phases were degassed and filtered by being passed through 0.22 µm filters (Millipore, Bedford, MA, USA) under vacuum.

### 4.3. Standard and Sample Preparations

The BRS−BoNTA and lyophilized pharmaceutical preparations were reconstituted according to the labeled potencies with purified water, following Pharmacopoeial procedures, in order to obtain concentrations of 100 U mL^−1^. For SEC and RPC, the working solutions were prepared to contain 50 and 25 U mL^−1^ of BoNTA, respectively. For the in vivo and in vitro bioassays, the solutions were reconstituted and diluted with 0.9% (*v*/*v*) sodium chloride to obtain doses between 6.90 and 12.10 U mL^−1^, and with RPMI 1640 to give concentrations ranging from 3 to 81 U mL^−1^, respectively.

### 4.4. Chromatographic Procedures

SEC Parameters

The SEC method was developed and validated, and the analyses were performed on a TSKgel^®^ G3000 SW_XL_ column (5 µm, 250 Å, 300 mm × 7.8 mm) from Tosoh Bioscience (Tokyo, Japan), protected with a guard column, and the elution was monitored using PDA set at 220 nm. A total of 50 µL was eluted isocratically with a mobile phase of a 50 mM L^−1^ potassium phosphate solution at pH 7.0, at a flow rate of 1.0 mL min^−1^. The temperature was set at 25 °C.

RPC Parameters

The RPC method was accomplished on a Zorbax 300 SB C_18_ column (5 µm, 300 Å, 150 mm × 4.6 mm) from Agilent (Santa Clara, CA, USA), maintained at 45 °C, following the procedure previously described [28]. The mobile phase consisted of a 50 mM L^−1^ sodium phosphate solution at pH 2.8, and acetonitrile (80:20, *v*/*v*), at a flow rate of 0.3 mL min^−1^, with detection using PDA set at 214 nm.

### 4.5. Bioassays

T-47D cell culture bioassay

The in vitro bioassay was carried out as described elsewhere [18,28], modified accordingly. The T-47D cells (ATCC HTB−133) were seeded at a density of 3 × 10^5^ cells mL^−1^, and the assay was performed with doses of BoNTA, between 3 and 81 U mL^−1^, in triplicate. The responses were assessed using 10 µL of alamarBlue^TM^. The absorbances were read at 570 and 600 nm, and the CombiStats^TM^ software, licensed by the European Directorate for the Quality of Medicines & HealthCare (EDQM) of the Council of Europe (Strasbourg, France), was used to calculate the biological activity using the parallel-line statistical method.

LD_50_ mouse bioassay

Female Swiss mice weighing between 23–27 g were randomly assigned to the BRS−BoNTA, pharmaceutical preparations, and control groups, with 10 mice per treatment group. Five concentrations with 1.15-fold dilution series between 6.90–12.10 U mL^−1^ were selected, and the bioassay was carried out as described previously [10,28], optimized. The responses of the mortality were transformed to probits, and the potency with confidence intervals (*p* = 0.05) was calculated using the CombiStats^TM^ software. All of the procedures used in the present study were approved by the Ethics Committee on Animal Use of the Federal University of Santa Maria (protocol number 8227050716, 23 September 2016).

In vitro cytotoxicity test

The in vitro neutral red uptake assay was performed as described elsewhere [36], with exposure of the cell lines NCTC Clone 929 (ATCC CCL−1) and CHO-K1 (ATCC CCL−61) to intact and degraded products. The cells’ viability was measured by a color reaction, and recorded their absorbance at 540 nm.

### 4.6. Validation of the SEC Method

The method was validated using pharmaceutical preparations with a label claiming 100 U mL^−1^, and the parameters of specificity, linearity, LD, LQ, precision, accuracy, robustness, and system suitability were assessed according to the international guidelines [37,38].

Specificity

The specificity was evaluated analyzing a BRS−BoNTA solution and a pharmaceutical preparation (100 U mL^−1^) stressed at 60 °C for 8 h, followed by shaking for 1 h. Then, they were diluted with purified water to 50 U mL^−1^. In addition, a possible interference from the excipients of the formulation was determined by analyzing the sample identified as placebo (in-house mixture of HSA and sodium chloride in the same concentration of the pharmaceutical preparations). The peak purity of BoNTA and the altered forms were also checked by superimposing the spectra corresponding to the upslope, apex, and downslope using a PDA detector.

### 4.7. Analysis of BoNTA in Biopharmaceutical Products

To assess the content/potency of the biotherapeutics, and to evaluate the capability of the methods, seven batches from different manufacturers, including Botox^®^, Botulim^®^, and Botulift^®^, which are commercially available for clinical use, were purchased. The results of the analyses were calculated as percentage recoveries against BRS−BoNTA.

## Figures and Tables

**Table 1:** Accuracy of the size-exclusion chromatography (SEC) for determining botulinum neurotoxin type-A (BoNTA) in biopharmaceutical formulations.

Nominal Concentration (U mL^−1^)	Mean Concentration Measured *^a^* (U mL^−1^)	RSD *^b^* (%)	Accuracy *^a^* (%)	Bias *^c^* (%)
40	40.02	0.73	100.04	0.05
50	50.13	0.14	100.26	0.26
60	60.56	0.22	100.93	0.93

*^a^* = Mean of the three replicates. *^b^* = relative standard deviation (RSD). *^c^* = Bias = [(measured concentration − nominal concentration)/nominal concentration] × 100.

**Table 2:** Chromatographic parameters and range evaluated during robustness testing using the one-variable-at-a-time procedure for the SEC method.

Variable	Range Investigated	BoNTA *^a^* (%)	RSD *^b^* (%)	Optimized Value
Mobile phase pH	6.5	99.56	0.57	7.0
	7.0	101.12	0.17	
	7.5	100.13	0.86	
Potassium phosphateconcentration (mM)	30	99.86	0.54	50
	50	100.19	0.29	
	70	102.40	0.43	
Flow rate (mL min^−1^)	0.8	99.90	0.69	1.0
	1.0	100.16	0.19	
	1.2	98.72	0.98	
Wavelength (nm)	210–320	−	−	220

*^a^* = Mean of the three replicates. *^b^* = RSD, relative standard deviation.

**Table 3:** Comparative content/potency assessment of BoNTA in biopharmaceutical formulations using the LD_50_ mouse bioassay, T-47D cell bioassay, and liquid chromatography (LC) methods.

Sample	Theoretical Amount	LD_50_ mouse bioassay *^a^*		T-47D bioassay *^a^*		SEC *^a^*		RPC *^a^*	
		Potency	Confidence Intervals	Potency	Confidence Intervals	Monomer	HMW Proteins	Main Peak	Related Proteins
	(U mL^−1^)	(%)	(*p* = 0.95)	(%)	(*p* = 0.95)	(%)	(%)	(%)	(%)
1	100	88.70	79.70–98.20	90.50	84.20–97.30	89.10	0.52	87.90	0.41
2	100	94.70	86.60–103.50	95.10	88.80–101.80	95.50	0.50	94.20	0.12
3	100	113.10	102.80–124.90	114.30	108.80–124.50	115.10	0.15	113.56	0.08
4	200	98.70	87.40–111.30	99.90	90.20–110.70	99.02	0.21	100.10	0.05
5	100	94.60	84.50–105.40	95.30	87.70–103.40	94.25	0.72	93.40	0.52
6	100	101.50	90.19–114.65	102.30	92.10–113.70	101.92	0.16	100.66	0.18
7	100	98.40	88.30–109.60	99.30	90.80–108.60	99.31	0.13	98.30	0.07
Mean *^a^*	−	98.46	−	99.53	−	99.17	0.34	98.30	0.23
SD *^b^*	−	7.72	−	7.58	−	8.17	0.24	8.07	0.18
							ANOVA		F calculated
							Between-methods		0.02976

*^a^* = Mean of the three replicates. *^b^* = standard deviation (SD). ANOVA—analysis of variance.

**Table 4:** Comparative content/potency assessment of BoNTA of the intact sample and after stress condition using the bioassays and LC methods.

Sample	Theoretical Amount	LD_50_ Mouse Bioassay *^a^*		T–47D Bioassay *^a^*		SEC *^a^*		RPC *^a^*	
		Potency	Confidence Intervals	Potency	Confidence Intervals	Monomer	HMW Proteins	Main Peak	Related Proteins
	(U mL^−1^)	(%)	(*p* = 0.95)	(%)	(*p* = 0.95)	(%)	(%)	(%)	(%)
4 *^b^*	200	98.70	87.40–111.30	99.90	90.20–110.70	99.02	0.21	100.10	0.05
4 *^c^*	−	50.90	42.60–61.20	52.30	43.20–60.80	53.40	14.19	50.70	19.45
4 *^d^*	−	151.20	143.70–159.10	153.80	145.90–160.20	0152.10	0.12	149.80	0.10
4 *^e^*	−	Inactive	−	29.10	22.10–36.90	77.90	8.62	−	−
4 *^f^*	−	95.20	87.00–103.00	97.40	89.40–105.30	−	−	80.53	10.26

*^a^* = Mean of the three replicates. *^b^* = Intact sample. Content of the altered samples: *^c^* = 50%, *^d^* = 150%, *^e^* = stressed conditions, and *^f^* = UV degradation.
